# Age-related impairments in active learning and strategic visual exploration

**DOI:** 10.3389/fnagi.2014.00019

**Published:** 2014-02-14

**Authors:** Kelly L. Brandstatt, Joel L. Voss

**Affiliations:** Department of Medical Social Sciences, Ken & Ruth Davee Department of Neurology, and Interdepartmental Neuroscience Program, Feinberg School of Medicine, Northwestern UniversityChicago, IL, USA

**Keywords:** active learning, memory, aging, age-related memory impairment, vicarious trial-and-error behavior, hippocampus, prefrontal cortex, revisitation

## Abstract

Old age could impair memory by disrupting learning strategies used by younger individuals. We tested this possibility by manipulating the ability to use visual-exploration strategies during learning. Subjects controlled visual exploration during active learning, thus permitting the use of strategies, whereas strategies were limited during passive learning via predetermined exploration patterns. Performance on tests of object recognition and object-location recall was matched for younger and older subjects for objects studied passively, when learning strategies were restricted. Active learning improved object recognition similarly for younger and older subjects. However, active learning improved object-location recall for younger subjects, but not older subjects. Exploration patterns were used to identify a learning strategy involving repeat viewing. Older subjects used this strategy less frequently and it provided less memory benefit compared to younger subjects. In previous experiments, we linked hippocampal-prefrontal co-activation to improvements in object-location recall from active learning and to the exploration strategy. Collectively, these findings suggest that age-related memory problems result partly from impaired strategies during learning, potentially due to reduced hippocampal-prefrontal co-engagement.

## INTRODUCTION

Older adults frequently perform poorly on memory tests. However, it is unclear to what extent these problems result from memory impairment *per se*, from problems with using learning strategies that support memory in younger individuals, or from some combination of these factors. For instance, source/relational memory deficits in older adults have been related to less engagement of relational encoding strategies that are used more frequently and spontaneously by younger individuals (Naveh-Benjamin et al., 2007), and that are likely mediated in part by prefrontal cortex (PFC). Structural changes in hippocampus and surrounding cortex of the medial temporal lobe (MTL) known to be critical for long-term memory have been identified in older individuals, yet changes are also consistently identified in cortical areas, including especially PFC (Raz et al., 2005; Giorgio et al., 2010). Indeed, some evidence indicates that strategic functions mediated by PFC are impaired by aging (e.g., Velanova et al., 2006), and that preservation of strategic capabilities in aging is a protective factor against age-related memory decline (Park and Reuter-Lorenz, 2009). Furthermore, PFC-mediated monitoring of memory signals can be used by younger individuals to allocate resources to important information during study, and this ability is potentially disrupted by aging (Isingrini et al., 2008). However, it remains unclear whether age-related strategy impairments derive from poor PFC-mediated monitoring and control, poor MTL-mediated memory signals, a combination of these impairments, or other factors such as global age-related cognitive decline.

A variety of age-related changes in the use of strategies have been identified for several cognitive domains (Lemaire, 2010). In the domain of learning and memory, most studies have focused on strategies used during verbal-learning paradigms (e.g., memory for material such as word lists and word pairings). For example, Naveh-Benjamin et al. (2007) found that young adults often spontaneously engage in semantically “deep” encoding and thereby demonstrate better subsequent memory performance for word lists (e.g., Craik and Tulving, 1975). In contrast, older adults used this strategy less frequently and thus performed relatively poorly on the memory test. However, older adults could be encouraged to adopt this strategy in order to improve performance. Aging has also been shown to impair awareness of memory success or failure (i.e., “meta-cognitive” awareness or “meta-memory,” e.g., (Isingrini et al., 2008; Wong et al., 2012), and therefore strategies involving meta-memory could be impaired. In younger individuals, meta-memory capabilities allow the learner to use knowledge of her own learning successes and failures to direct attention to material that needs additional resources, thus improving learning and subsequent memory performance (Metcalfe, 2009). In this sense, the strategic exploration of key information can boost learning in young adults, and potentially also in older adults. Indeed, when explicitly told which word pairs are relatively easy versus relatively difficult to learn, both younger and older adults use this information to select the items that require additional study (Price et al., 2010). Some evidence for a deficit in using self-assessments of memory to guide strategies has come from studies of skill learning in which subjects must learn to find word pairs in a lookup table, and strategies can include either rapid scanning of the table or memory-based retrieval of learned pairings. For instance, Touron (2006) found greater inconsistency in shifting between scanning-based and memory-based strategies for older versus younger adults that were not secondary to poor memory (as determined by item-level analysis), suggesting relative impairment in using self-assessments of memory to guide effective strategies (see also Touron et al., 2011).

Thus, relatively little is known regarding how aging influences the ongoing self-assessment of memory to guide effective strategies. This could be particularly critical for exploration, as this requires constant assessments. Furthermore, we argue that focusing experiments on strategies used during exploration will not only be useful in advancing the human studies, but this line of inquiry will also promise to have strong translational potential in terms of linking human age-related cognitive deficits to the animal literature. Indeed, Metcalfe and Jacobs (2010) have described a variety of strategies based on the strategic exploration of information during learning that can be observed in the behavior of humans as well as nonhuman animals in a variety of circumstances. Virtually nothing is known about how aging impairs the memory advantages gained through the strategic exploration of crucial information during learning (i.e., aside from verbal-learning paradigms). This information could be important for linking knowledge of neural mechanisms of memory decline across human and nonhuman animal models (Mata et al., 2013). Although standard memory tests involving verbal materials and self-report measures of performance are useful for understanding age-related memory impairment and have some parallels with tests used in animal models (Alexander et al., 2012), strategic exploratory behaviors provide attractive targets for translational study given that they do not require language-specific capabilities that are unique to humans.

**FIGURE 1:**
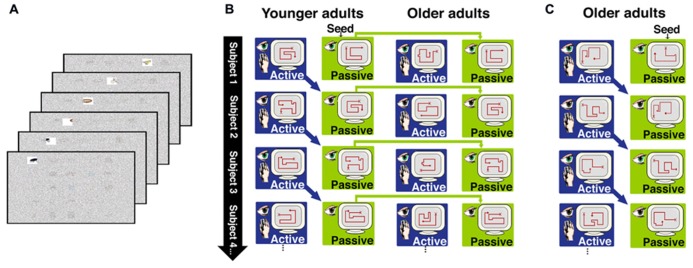
**Overview of the behavioral paradigm for Experiments 1 and 2.** **(A)** In both experiments, subjects studied objects arranged in a 5 × 5 grid. Objects were viewed through a window that permitted clear viewing of only one object at a time. The figure depicts the window moving across the top row of objects, to reveal an insect, and ring, a bicycle, and a bird. Window movements were controlled by the subject in the active learning condition, and were prerecorded from the previous subject and merely watched in the passive learning condition, as described in the text. **(B)** In Experiment 1, passive window movements were yoked to active window movements within younger subjects, and the passive window movements from younger subjects were also delivered to older subjects. Older and younger subjects controlled window movements in the active condition. **(C)** In Experiment 2, active and passive window movements were yoked within older subjects only.

We therefore sought to identify effects of aging on memory benefits obtained from the strategic control of ongoing exploratory behavior during learning. Older and younger adults were tested using a paradigm that we previously developed to manipulate the ability to use exploratory strategies during learning and to assess the effects of these strategies on subsequent memory performance (Voss et al., 2011a,b,c). Subjects studied displays of object images through a “window” that permitted clear viewing of only one object at a time (**Figure 1A**). In an active study condition, subjects used a computer mouse for online control of the viewing window movements. In contrast, window movements were predetermined in a passive study condition. We have previously demonstrated robustly superior memory for objects and their studied locations following active compared to passive study (Voss et al., 2011a,b,c), with this “active learning advantage” presumably resulting from the strategic control of visual exploration that is provided in the active but not the passive condition.

Furthermore, we have previously identified one specific exploration strategy that occurs when individuals immediately look back to restudy objects for a second time using a back-and-forth viewing pattern, termed the “revisitation” strategy (Voss et al., 2011b,c). Revisitation improved memory for the specific objects studied in this manner, but only when subjects actively generated revisitation in the active condition, not when the same pattern was merely viewed in the passive condition. In the current experiments, we therefore sought to identify effects of aging on the general memory benefits conferred by active control of exploratory behavior during study, as well as on the generation of revisitation strategy and its benefits to memory. Our previous identifications of brain regions correlated with and necessary for specific instances of distinct active learning advantages in young adults (Voss et al., 2011a,b) permit inferences regarding the neural substrates of age-related impairments based on patterns of behavioral impairment in older adults.

In Experiment 1, we tested older and younger adults using the aforementioned paradigm (**Figure 1**) in order to identify age-related differences in the active learning advantage and in the revisitation strategy. In the passive condition, window movements were predetermined based on the active movements made by subjects in the active condition (using a subject-to-subject “yoking” procedure; **Figure 1B**). This ensures that the same information is provided in both the active and passive study conditions. For Experiment 1, the prerecorded patterns of window movements were taken from the active condition of younger subjects and used for the passive condition of both younger and older subjects. Thus, the same visual information was provided in the passive condition for younger and older subjects, and therefore subsequent memory performance could be compared across age groups in order to determine if aging influences memory following passive study (i.e., without any exploration strategies used in either age group). In Experiment 2, window movements in the passive condition were taken from the active condition of older subjects, in order to determine if the nature of passive window movements (i.e., taken from an older subject versus taken from a younger subject) had any influence on the performance of older subjects.

## MATERIALS AND METHODS

### EXPERIMENT 1

Data were collected from 20 older adults (age 60–73, mean = 65 years) and 20 younger adults (age 18–24, mean = 20 years). All subjects had normal or corrected-to-normal vision. Older adults were screened for possible dementia using the Mini Mental State Exam (MMSE). MMSE scores were in the normal range of 24 or above (mean = 28.5, range = 25–30), indicative of healthy aging (Crum et al., 1993).

Participants studied sets of 25 objects arranged on a 5 × 5 grid displayed on a monitor, each for 60 s. Objects were selected from a set of common, readily nameable, color pictures (Roission and Pourtois, 2004). A semi-transparent noise mask obstructed all objects from view except through a window that allowed clear viewing of one object at a time (**Figure 1A**). Subjects were instructed to memorize all objects and their locations in anticipation of the upcoming memory tests. All subjects participated in both active and passive study conditions, with three distinct 25-object grids studied actively and three passively, in interleaved and counterbalanced order. In the active study condition, subjects were able to control the viewing window from moment-to-moment using a computer mouse, thus providing full control of the order and duration of study. The window movements were predetermined in the passive study condition, and participants merely viewed what was shown to them. In Experiment 1, the active window movements for younger subject *n* were recorded and played back as the passive movements to younger subject *n* + 1 and to the corresponding older subject (**Figure 1B**). This method ensured that the very same objects were viewed in the same order for the same durations in the active and passive conditions for younger adults. Furthermore, the same objects were viewed in the same order and for the same durations in the passive condition for the younger versus older adults. For the first subject only, movements of the viewing window for the passive condition were taken from the active movement record of an additional subject (i.e., a “seed” record), who did not participate in memory tests or contribute any other data to analyses.

Window movements were recorded continuously at 60 Hz and analyzed offline. A computer algorithm created a time-series of visited objects based on the continuous record. Objects that were studied for a total duration of less than 200 ms were excluded from all analyses (~1% of objects). An algorithm was used to score revisitation study strategy. Any individual viewing periods on an object less than 60 ms in duration were excluded from analysis of revisitation to guard against influences from partial/spurious views. The algorithm coded all back-and-forth viewing involving between two and six objects (e.g., A–B–A to A–B–C–D–E–F–E–D–C–B–A) as “revisitation,” and all other viewing as “other.” Longer revisitation sequences (i.e., those involving seven or more objects) were not considered because they rarely occurred. Because object-to-object transitions almost always occurred in diagonal and horizontal paths, spontaneous revisitation rarely occurred with more geometrically complicated paths (e.g., A–B–C–A), and these were therefore scored as “other.” Algorithm codification of spontaneous revisitation was confirmed for each subject by visual inspection of recreated viewing paths. Some subjects did not generate any revisitation, and therefore not all subjects contributed data to analyses of memory performance as a function of revisitation (2 older subjects excluded in Experiment 1, and 3 and 4 older subjects excluded from the active and passive conditions, respectively, in Experiment 2).

After studying six 25-object grids, half actively and half passively, subjects were given two memory tests in the following order: (i) spatial recall of item location (25 actively and 25 passively studied objects, randomly selected) and (ii) yes/no recognition of repeat versus novel items (for all objects not used in the spatial test). In the spatial recall test, subjects positioned studied objects individually onto an empty grid with markers indicating the 25 locations where objects were located during study. In the item recognition test, studied items were shown one at a time, randomly intermixed with an equal number of unstudied (i.e., new) items. Subjects made old/new recognition judgments to each item while simultaneously rating confidence on a four-point scale: confident old, unsure old, unsure new, and confident new. Our primary analyses of spatial recall performance use the distance between the object’s studied location and where it was positioned by the subject during the test (placement error, cm). We also quantified the proportion of “direct hits,” when the object was repositioned exactly where it was studied. The statistical analyses yielded the same patterns of significance for this measure as for placement error (at the *P* < 0.05 significance threshold), and so only placement error is reported. Our primary analyses of item recognition performance use discrimination sensitivity (d′), a normalized measure of correct endorsement of old items (hits) minus incorrect endorsement of new items (false alarms), collapsed across confidence ratings. Statistical analyses utilized repeated-measures ANOVA (RM-ANOVA) as well as planned pairwise comparisons.

### EXPERIMENT 2

Data were collected from a new sample of 20 older adults (age 64–77, mean = 67 years). All subjects had normal or corrected-to-normal vision. MMSE scores were in the normal range for all older adults (mean = 28.8, range = 26–30), indicating healthy aging.

The testing procedures were identical to Experiment 1 except that window movements in the passive condition were derived from the active condition of older subjects. That is, the active window movements for subject *n* were recorded and played back as the passive movements to subject *n* + 1 (**Figure 1C**). This method ensured that the very same objects were viewed in the same order for the same durations in the active and passive conditions for older adults. The primary analysis objective was to determine whether older adults benefited from active study under these conditions and, if so, for which of the two test formats. We used planned comparisons to identify these active learning benefits and to weigh them against the active learning benefits identified in Experiment 1. Young adults were not included (i.e., window movements from older adults were not given to younger adults for the passive condition), given that our goals were to identify factors that could have impaired the performance of older adults relative to younger adults in Experiment 1, not on factors that can modulate performance of younger adults. Furthermore, our previous findings suggest that the nature of the passive condition has little influence on performance for younger adults (Voss et al., 2011a), and indeed the performance of young adults in the Experiment 1 passive condition was nearly identical to performance we have previously observed in those experiments.

## RESULTS

### EXPERIMENT 1

#### Active versus passive study in older and younger adults

**FIGURE 2:**
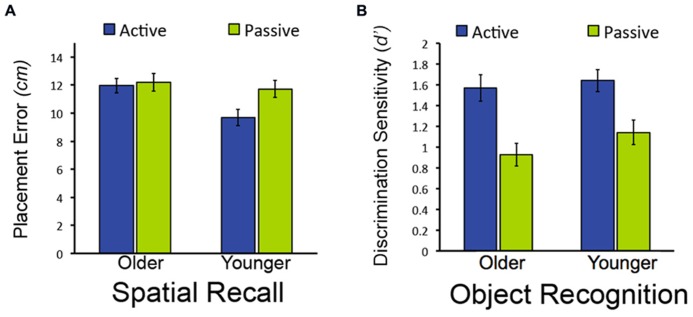
**Effects of age on object-location recall but not object recognition in Experiment 1. (A)** Mean placement error for object-location recall in Experiment 1. **(B)** Mean performance (discrimination sensitivity) for object recognition in Experiment 1. Note that lower bars indicate better performance for object-location recall (less placement error), whereas higher bars indicate better performance for object recognition (higher discrimination sensitivity). Error bars depict standard error.

We first sought to determine whether memory performance would differ for older and younger adults for items studied in the passive condition, when neither group could use exploratory learning strategies. To the extent that aging causes memory impairment, older adults would be expected to perform worse than younger adults. However, if age-related memory impairments originate instead from the reduced use of strategies in older versus younger adults, then both groups would be expected to perform similarly following passive study. Both memory tests provided evidence for the latter. For the spatial recall test, there was no reliable difference between old and young adults in spatial recall error for objects studied in the passive condition [*t*_(38)_ = 0.52, *P* = 0.604; **Figure 2A**]. Likewise for item recognition, there was no significant difference between older and younger adults’ correct discrimination of old from new objects for old objects studied in the passive condition [*t*_(38)_ = 1.33, *P* = 0.192; **Figure 2B**; Confidence ratings are provided in **Table 1**]. Thus, older and younger adults performed similarly on both test formats for items studied passively.

**Table 1:** Mean and standard error for object recognition confidence ratings in Experiment 1 and Experiment 2.

	Active studied (old)				Passive studied (old)				New			
	HC old	LC old	LC new	HC new	HC old	LC old	LC new	HC new	HC old	LC old	LC new	HC new
Experiment 1 older	0.72 (0.03)	0.08 (0.02)	0.09 (0.02)	0.10 (0.02)	0.52 (0.02)	0.09 (0.02)	0.16 (0.02)	0.23 (0.03)	0.18 (0.02)	0.10 (0.02)	0.22 (0.03)	0.50 (0.05)
Younger	0.67 (0.03)	0.10 (0.02)	0.12 (0.02)	0.11 (0.02)	0.48 (0.04)	0.12 (0.01)	0.16 (0.03)	0.23 (0.04)	0.11 (0.01)	0.10 (0.01)	0.29 (0.04)	0.50 (0.05)
Experiment 2 older	0.64 (0.06)	0.09 (0.02)	0.09 (0.02)	0.18 (0.05)	0.53 (0.05)	0.09 (0.02)	0.14 (0.03)	0.24 (0.05)	0.23 (0.04)	0.09 (0.02)	0.20 (0.04)	0.48 (0.05)

*Response types included high-confidence old (HC Old), low-confidence old (LC Old), low-confidence new (LC New), and high-confidence new (HC New).
*

In contrast, older and younger subjects differed in terms of the memory benefits derived from active versus passive study. For the spatial recall test, a marginal interaction between study condition (active/passive) and age (young/old) indicated that the performance difference between actively and passively studied items did not differ for younger compared to older adults [*F*_(1,38)_ = 3.98, *P* = 0.053; $\eta_{\rho }^{2}$ = 0.095; main effect of study condition: *F*_(1,38)_ = 6.12, *P* = 0.018, $\eta_{\rho }^{2}$ = 0.139; main effect of age: *F*_(1,38)_ = 3.83, *P* = 0.058, $\eta_{\rho }^{2}$ = 0.092]. There was significantly less spatial recall placement error for active-studied versus passive-studied objects for younger subjects [*t*_(19)_ = 3.40 *P* = 0.003], but not for older subjects [*t*_(19)_ = 0.32, *P* = 0.754; **Figure 2A**]. Likewise, spatial recall placement error was reliably less in younger subjects compared to older subjects [*t*_(38)_ = 2.90, *P* = 0.006]. This indicates that active learning was beneficial to spatial memory performance relative to passive learning in younger but not older adults, consistent with the hypothesis that younger adults use strategies that aid learning to a greater extent than older adults.

However, the same was not true of performance on the item recognition test. For this test, active study benefited older as well as younger adults’ performance, with benefits of similar magnitude for both groups. Discrimination of old from new objects was significantly better for objects studied in the active condition versus the passive condition for both older and younger subjects [main effect of study condition: *F*_(1,38)_ = 62.24, *P* < 0.001, $\eta_{\rho }^{2}$ = 0.621], with no significant interaction of study type by group [*F*_(1,38)_ = 1.00, *P* = 0.324; main effect of age: *F*_(1,38)_ = 0.96, *P* = 0.332; **Figure 2B**]. Performance for actively studied objects did not differ for older versus younger adults [*F*_(1,38)_ = 1.00, *P* = 0.324]. Taken together with the results from the spatial recall test, these findings indicate that the effects of age on the benefits of active learning were selective. That is, age-related impairment of active learning benefits was evident for spatial recall, whereas older adults showed a similar active learning benefit as younger adults for object recognition.

#### Revisitation study strategy in older and younger adults

**FIGURE 3:**
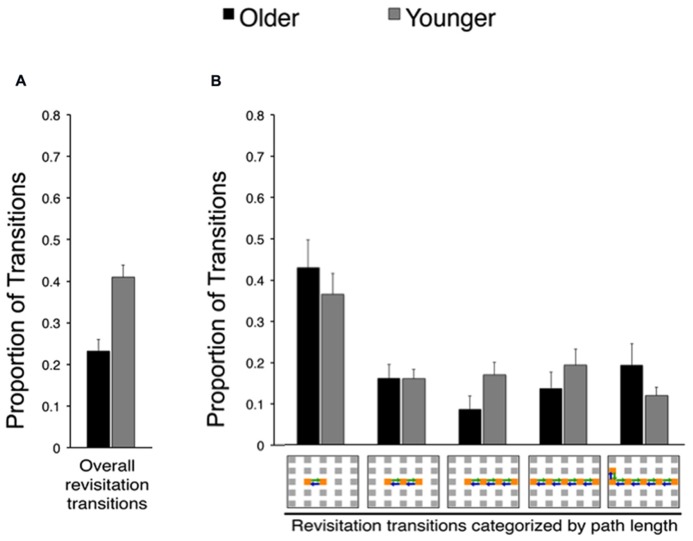
**Effects of age on spontaneous revisitation strategy in Experiment 1. (A)** The overall proportion of transitions categorized as involving spontaneous revisitation is shown for older and younger adults. **(B)** The overall proportion of transitions are broken down as a function of the number of objects within each revisitation event (two to six objects) on the right. This shows the distribution of revisitation “path lengths” in each age group, irrespective of overall differences in the amount of revisitation (i.e., the overall amount is given in Panel A, and Panel B shows the proportion of the total involved in each path length separately for each age group). Back-and-forth transitions are shown as arrows on the grid. Error bars depict standard error.

In order to identify age-related changes in the prevalence and benefits of revisitation strategy, we quantified the proportion of transitions that were involved in revisitation during active study (i.e., generated by each subject in his/her active condition) in younger and older adults. Younger adults generated more revisitation than older adults [**Figure 3**; *t*_(38)_ = 4.28, *P* = 0.002]. Although revisitation was less prevalent for older adults, the general characteristics of revisitation were approximately the same as for younger subjects. That is, when revisitation events were categorized according to how many object-to-object transitions were involved (from 2 to 6 objects; **Figure 3B**), there were no significant interaction of age (older, younger) with revisitation path length [2, 3, 4, 5, or 6 objects; *F*_(4,152)_ = 0.94, *P* = 0.440; main effect of age: *F*_(4.152)_ = 2.11, *P* = 0.154; main effect of path length: *F*_(4,152)_ = 10.96, *P* < 0.001, $\eta_{\rho }^{2}$ = 0.24]. Thus, older adults generated less revisitation than younger adults, but revisitation characteristics were approximately the same.

We next identified the effects of revisitation on subsequent memory performance. In the active condition, spatial recall performance for objects studied with revisitation was significantly better than for objects studied otherwise for younger subjects [*t*_(__19)_ = 3.66, *P* = 0.001; **Table 2**]. However, this was not the case for the objects that younger subjects merely watched being studied with revisitation in the passive condition [*t*_(19)_ = 0.35, *P* = 0.732]. In contrast, spatial recall in older subjects did not benefit from revisitation in either the active or passive conditions [respectively, *t*_(18)_ = 0.22, *P* = 0.829 and *t*_(18)_ = 0.88, *P* = 0.390]. These results suggest that the act of generating revisitation (in the active condition) did not reliably improve older adults’ spatial recall, as it did for younger adults. Importantly, for younger adults, the beneficial effects of revisitation were selective for the active condition, with no benefit when the revisitation pattern was viewed in the passive condition, indicating that the beneficial effects of revisitation in younger subjects were specific to when this visual exploration pattern was generated as a learning strategy.

**Table 2:** Mean and standard error for object recognition discrimination sensitivity (*d*^′^) for objects studied with revisitation versus other-studied objects in Experiment 1 and Experiment 2.

	Active		Passive	
	Revisitation	Other	Revisitation	Other
Experiment 1 older	2.3 (0.4)	1.4 (0.2)	0.5 (0.2)	0.7 (0.4)
Younger	2.2 (0.4)	1.3 (0.2)	1.1 (0.4)	0.6 (0.3)
Experiment 2 older	2.2 (0.4)	1.4 (0.3)	0.4 (0.5)	1.0 (0.3)

In the active condition, item recognition (*d*′) for objects studied with revisitation was significantly better than for objects studied otherwise for younger subjects [*t*_(19)_ = 2.96, *P* = 0.008; **Table 3**]. In contrast, there was no performance difference for revisitation-studied versus other-studied in the passive condition (t_(19)_ = 1.36, P = 0.190). Unlike the pattern identified for spatial recall performance, older subjects also benefited from active revisitation. Performance for objects studied with revisitation was significantly better than for objects studied otherwise in the active condition [*t*_(17)_ = 2.20, *P* = 0.042], but not in the passive condition [*t*_(19)_ = 0.87, *P* = 0.396].

**Table 3:** Mean and standard error for object-location recall placement error for objects studied with revisitation versus other-studied objects in Experiment 1 and Experiment 2.

	Active		Passive	
	Revisitation	Other	Revisitation	Other
Experiment 1 older	205.8 (9.5)	207.1 (14.6)	209.8 (11.9)	222.3 (14.2)
Younger	157.3 (10.9)	188.0 (10.7)	209.6 (13.9)	204.5 (13.0)
Experimet 2 older	204.9 (10.5)	207.7 (9.3)	230.4 (17.4)	212.9 (12.9)

#### Relationships between age and performance for older adults

It is important to note that the group of older adults included a 13-year age range, and performance could have differed meaningfully within the group (in contrast, the group of younger adults included only a 6-year age range). To test for relationships between age and performance in the older adults, we performed correlations between age and active learning benefits (the active minus passive difference in performance for item recognition and spatial recall), between age and the amount of revisitation generated, and between age and performance for objects studied with revisitation versus objects studied otherwise. No correlations were significant (*r* values -0.09–0.27, *P* values 0.13–0.46), indicating that there was no significant variation in performance due to age within the older adult group.

#### Results summary for experiment 1

To summarize the results from Experiment 1, older adults were specifically impaired in using active exploration strategies to enhance learning of object-location information relative to younger adults. This deficit is striking in contrast to the relatively matched performance of older and younger adults when both groups are deprived of strategies in the passive study condition. However, it is notable that the movements of the viewing window in the passive condition for both younger and older subjects were derived from the active exploration of younger subjects. Thus, these age-related deficits could have been influenced by detrimental effects of viewing the exploration of younger subjects for the performance of older subjects. That is, older adults could differ from younger adults in terms of the speed of window movement, the frequency of movement, the frequency of revisitation, and other factors. Indeed, some evidence suggests that disrupting individuals’ preferred viewing style could be detrimental for performance (Chan et al., 2011). In our experiment, older and younger adults did not differ in performance following passive study, suggesting that any such differences in preferred window movements did not directly have negative impacts on the memory performance of older subjects. Nonetheless, because the experiment involved multiple interleaved active and passive study blocks, older subjects could have nonetheless learned to emulate the characteristics of the window movement patterns from younger subjects, with a potential detrimental effect on their exploration patterns in the active condition. In order to test this possibility, we used window movements recorded from other older adults for the passive condition in Experiment 2.

### EXPERIMENT 2

#### Active versus passive study in older adults

**FIGURE 4:**
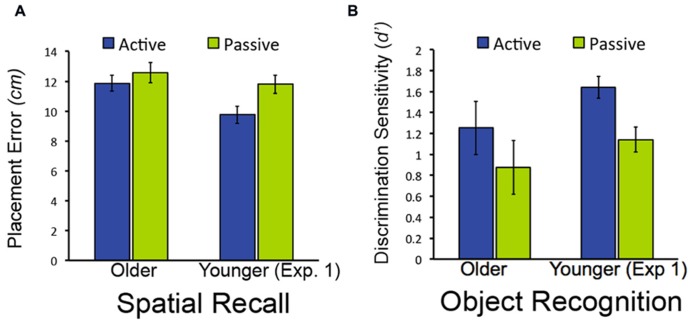
**Object-location recall and object recognition in Experiment 2.** **(A)** Mean placement error for object-location recall in Experiment 2. **(B)** Mean performance (discrimination sensitivity) for object recognition in Experiment 1. Data from younger subjects from Experiment 1 are shown to facilitate comparison with **Figure 2**. Note that lower bars indicate better performance for object-location recall (less placement error), whereas higher bars indicate better performance for object recognition (higher discrimination sensitivity). Error bars indicate standard error.

As was the case in Experiment 1, discrimination of old from new objects was superior in older adults for active relative to passive study [*t*_(19)_ = 2.37, *P* = 0.028; **Figure 4**; confidence ratings provided in **Table 1**]. Also as for Experiment 1, older adults did not demonstrate better spatial recall performance for active versus passive study [*t*_(__19)_ = 1.28, *P* = 0.216]. We also compared performance for older subjects in Experiment 2 versus Experiment 1. For both spatial recall and item recognition, performance in the passive condition did not differ for the older subjects in Experiment 2 versus the older adults in Experiment 1 [*t*_(38)_ = 0.34, *P* = 0.733 and *t*_(38)_ = 0.17, *P* = 0.865, respectively]. Likewise, performance in the active condition did not differ for older subjects in Experiment 2 versus Experiment 1 for both spatial recall and item recognition [*t*_(38)_ = 0.22, *P* = 0.830 and *t*_(38)_ = 1.11, *P* = .297, respectively). The magnitude of the advantage for active study compared to passive study for item recognition in older adults did not differ significantly for Experiment 1 versus Experiment 2 [*t*_(38)_ = 1.46, *P* = 0.152]. These results replicate the selective advantage for item recognition due to active learning in older adults from Experiment 1, and indicate that origin of the passive window movements (i.e., from older versus from younger subjects) did not significantly influence performance for older adults for either the active or passive conditions.

#### Revisitation study strategy in older adults

**FIGURE 5:**
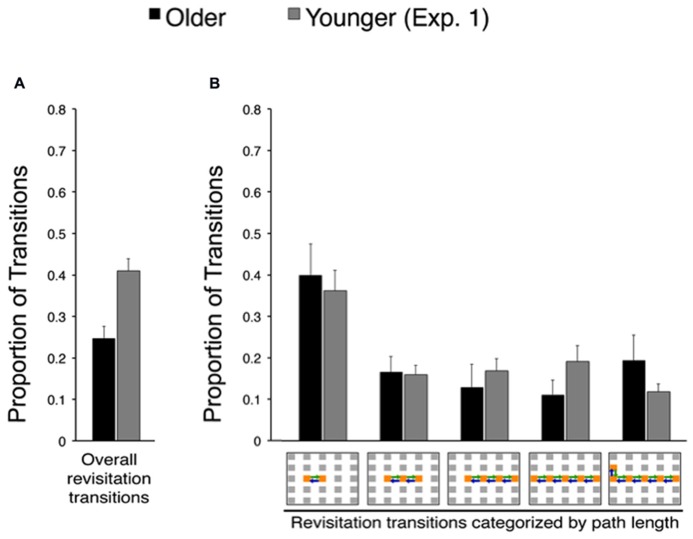
**Spontaneous revisitation strategy in Experiment 2. (A)** The overall proportion of transitions categorized as involving spontaneous revisitation. **(B)** The overall proportion of transitions are broken down as a function of the number of objects within each revisitation event (two to six objects), as in **Figure 3B**. Back-and-forth transitions are shown as arrows on the grid. Data from younger subjects from Experiment 1 are shown to facilitate comparison with **Figure 3**. Error bars depict standard error.

Revisitation was also similar for older adults in Experiment 2 compared to older adults in Experiment 1. **Figure 5** shows the proportion of revisitation generated by older adults in Experiment 2 plotted against revisitation generated by younger adults in Experiment 1, so that the same effects versus younger adults identified in Experiment 1 can be readily observed. There was no significant difference in the overall amount of revisitation generated in the active condition for older adults in Experiment 2 versus Experiment 1 [*t*_(38)_ = 0.36, *P* = 0.722]. Likewise, there was no significant interaction between experiment (1, 2) and the amount of revisitation for each path length (2, 3, 4, 5, or 6 objects) for older adults [*F*_(4,148)_ = 0.08, *P* = 0.989; main effect of experiment: *F*_(1,37)_ = 2.00 *P* = 0.165, main effect of path length: *F*_(4,148)_ = 8.52, *P* < 0.001, $\eta_{\rho }^{2}$ = 0.187], indicating similar revisitation characteristics for older adults in both experiments.

For older adults in Experiment 2, item recognition performance was marginally greater for objects studied with revisitation than for objects studied otherwise in the active condition [*t*_(16)_ = 1.89, *P* = 0.078], but unlike Experiment 1, there was also a small increase in performance due to revisitation in the passive condition [*t*_(16)_ = 2.31, *P* = 0.035; **Table 2**]. Indeed, the magnitude of the item recognition difference for revisitation-studied versus other-studied items in the active condition did not differ significantly for older adults in Experiment 2 versus Experiment 1 [*t*_(35)_ = 0.09, *P* = 0.933]. In contrast, spatial recall did not benefit from revisitation. Spatial recall performance did not differ for objects studied with revisitation versus otherwise for either the active or passive conditions [*t*_(17)_ = 0.11, *P* = 0.916, *t*_(15)_ = 1.33, *P* = 0.203, respectively; **Table 3**]. These results are consistent with those reported in Experiment 1, suggesting that impairments in exploration learning strategies in older adults were not due to the passive condition that was used in Experiment 1.

#### Relationships between age and performance for older adults

As for Experiment 1, we tested for relationships between age and performance in the older adults by performing correlations between age and active learning benefits (the active minus passive difference in performance for item recognition and spatial recall), between age and the amount of revisitation generated, and between age and performance for objects studied with revisitation versus objects studied otherwise. No correlations were significant (*r* values -0.23–0.34, *P* values 0.07–0.39), indicating that there was no significant variation in performance due to age within the older adult group.

## DISCUSSION

These findings support several novel conclusions regarding the nature of age-related changes in memory and in learning strategies. First, older adults demonstrated no memory impairments relative to younger adults following passive study. This was true for both item recognition and spatial recall, and is striking in light of the spatial recall and associative memory deficits commonly identified in older adults (e.g., Old and Naveh-Benjamin, 2008; Shing et al., 2010; Alexander et al., 2012). By limiting the possibility for active exploration strategies during learning in the passive condition, memory performance of older and younger adults became indistinguishable. This underscores the possibility that age-related memory decline can result from reduced utilization of strategies during study.

Like younger adults, older adults were able to benefit from active control of exploration during study. However, unlike in younger adults, these benefits were selective. Item recognition improved for active versus passive study in both older and younger adults, whereas only younger adults demonstrated superior spatial recall for active versus passive study. Thus, older adults were not able to improve their spatial recall performance via control of exploration during active study. It is notable that spatial recall performance in the passive condition did not differ for younger and older subjects. This selective inability to improve spatial recall by active exploration thus represents a deficit in the ability to strategically enhance object-location memory by active control of exploration in older adults.

Indeed, a specific active exploration strategy, revisitation, also differed in older adults compared to younger adults. Older adults engaged in revisitation less frequently than did younger adults. Furthermore, the benefits derived from revisitation differed for older compared to younger adults. In younger adults, item recognition and spatial recall were both superior for the objects studied with the revisitation strategy. Crucially, this revisitation benefit was selective for the active condition, when subjects generated the revisitation exploration pattern, whereas merely viewing the same pattern in the passive condition did not improve memory. Older adults also benefited from active revisitation, but only for item recognition. Revisitation did not improve spatial recall for older adults. This provides a specific instance of an age-related impairment in the utility of an exploration strategy for the improvement of object-location memory. These findings are consistent with the limited previous evidence that older adults have particular problems with strategies that improve associative memory performance (Naveh-Benjamin et al., 2007). In light of our previous evidence that hippocampal-dependent memory impairment virtually eliminates revisitation (Voss et al., 2011b), we interpret the revisitation strategy as an indication that individuals are using memory to guide the decision to revisit, thus strategically allocating more study time (i.e., attention) to objects that were not successfully encoded upon first viewing. We thus do not consider revisitation as a direct reflection of learning per se, but instead interpret it as a memory-based decision that correlates with learning success. Revisitation could be beneficial particularly because it directs attention to enable restudying as needed, which would be expected to enhance learning. It is unlikely that revisitation results from global factors such as poor working memory (i.e., individuals who forget things quickly must look back often to study again), given that such impairments would tend to produce higher levels of revisitation, whereas we found that older adults (who tend to have higher levels of global impairments in capabilities such as working memory) produced less revisitation. Moreover, revisitation did not differ in general characteristics such as the distribution of path length in younger versus older adults (**Figure 3B**), and thus likely indicated similar learning-related processes in younger and older adults when it was generated.

These results extend these previous findings on age-related impairments in learning strategies in several important regards. As reviewed above, earlier studies have suggested possible differences in using memory to select appropriate strategies (e.g., Touron, 2006) and impairments in memory confidence or “meta-memory” (e.g., Isingrini et al., 2008; Wong et al., 2012), but our study is unique in that effective strategies such as revisitation could be based on an ongoing assessment of memory. Our findings thus suggest that aging could involve reduced memory monitoring from moment to moment in order to guide exploration. In addition, unlike in previous studies, our use of the passive study condition provided a means of equating the lack of strategy use for younger and older adults, allowing us to determine that memory performance is matched when strategies are experimentally limited. Furthermore, we did not encourage older adults to adopt particular strategies during the active learning condition (as in Naveh-Benjamin et al., 2007), nor did we provide explicit feedback about items that would benefit from additional study (as in Price et al., 2010), but instead simply provided the opportunity by giving active control of visual exploration.

Older adults did adopt at least one strategy that could be quantified, the “revisitation” strategy (**Figure 3**; Voss et al., 2011b). This strategy was observed in subjects’ patterns of ongoing visual exploration, and thus did not involve self-report or other response demands to quantify. Although older subjects implemented this strategy, they did so to a lesser extent than did younger subjects and with less benefit for memory performance. It is important to note that the general features of revisitation were matched in younger and older adults, i.e., revisitation events included approximately the same distribution of item-to-item transitions in younger and older adults (**Figures 3B and 5B**). In contrast, severe deficits caused by hippocampal amnesia are associated not only with reduced overall prevalence of revisitation but also a qualitative shift in the number of transitions involved in each revisitation event (Voss et al., 2011b). Therefore, the current evidence is consistent with a reduction in production of revisitation, not in a qualitative shift in the nature of the revisitation strategy. Furthermore, revisitation did improve memory in older adults as it did in younger adults. However, as was the case for the general active learning benefit for older adults, the benefit they derived from revisitation was specific to item recognition memory, with no benefit to spatial recall. Although our results do not allow us to determine if memory deficits in aging could be ameliorated by further encouragement of helpful learning strategies, they do show that merely providing the opportunity for strategies by giving an active condition leads to the use of strategies by older adults, despite impairment in the prevalence of these strategies and in their benefit for associative memory relative to younger subjects.

Findings of age-related memory impairments are frequently consistent with the associative deficit hypothesis (Old and Naveh-Benjamin, 2008; Shing et al., 2010) suggesting that older adults have specific problems with memory for associative or relational material, with relatively intact item-specific memory. Interestingly, our findings were also consistent with an age-related distinction between items and associations, but in terms of the benefits of learning strategies. That is, in both experiments, active study improved item memory (recognition) but not associative memory (location recall) in older adults, whereas younger adults exhibited active learning benefits for both test formats. In contrast, older and younger subjects performed similarly for both test formats following passive study, when strategies were limited. This suggests that older adults had specific problems with using active learning strategies to improve associative memory. The specificity of these impairments to active learning benefits for one test format and not another provide evidence counter to the notion that age-related memory impairments are merely part of a nonspecific cognitive impairment that produces poor performance on many domains of testing (e.g., Siedlecki et al., 2005; Bugaiska et al., 2007). For instance, global age-related deficits such as cognitive slowing were unlikely responsible for the impairments we observed, given that self-paced control of study would have been expected to overcome impairments related to cognitive speed in older adults (and, furthermore, no impairment was evident for the passive condition, which would also be expected to show impairment based on slowing). The selective age-related impairment in improving associative learning in the active condition coupled with both the lack of associative memory deficit in the passive condition and with with the preserved ability to use revisitation to enhance item-memory performance is most consistent with the notion that older adults failed to monitor associative information to guide study choices. That is, reduced awareness of associative memory could have impaired the use of active strategies such as revisitation when they were needed to improve associative learning. This extends previous findings on associative memory deficits (e.g., Old and Naveh-Benjamin, 2008; Shing et al., 2010) and previous findings on meta-cognitive deficits (e.g., Wong et al., 2012) by suggesting that older adults have difficulties not with associative memory per se, but with monitoring associative memory in order to effectively implement strategies that would enhance associative memory if performed judiciously (and that can still be used to improve learning of other information, such as item learning).

Older adults’ selective impairment in using active learning strategies to improve location recall performance can be interpreted in light of our previous studies of neural substrates of active learning strategies in the current paradigm (Voss et al., 2011a,b,c). In healthy younger subjects, active study increased the correlation of hippocampal brain activity with activity in a variety of cortical regions relative to passive study, suggesting that beneficial effects of active study derive from the increased coordination of activity among hippocampus and other functionally specialized regions. Furthermore, this increase in activity correlation differentially predicted benefits of active study for item recognition versus location recall (Voss et al., 2011a). The degree to which active compared to passive study increased the activity correlation of hippocampus with lateral parietal cortex and ventral visual cortex (fusiform gyrus) correlated with the degree to which item recognition was improved by active versus passive study. In contrast, the same correlation was identified with respect to location recall, but involving hippocampus with lateral prefrontal cortex. Furthermore, the revisitation strategy correlated with activity of prefrontal cortex and hippocampus when it was generated in the active condition, but not the passive condition (Voss et al., 2011b). The specific deficits observed here in older subjects (no active benefit for location recall and reduced revisitation) therefore suggest that hippocampal-prefrontal transactions are compromised in aging. We also previously found that severe hippocampal damage in amnesic individuals eliminated any benefit from active learning and essentially abolished revisitation (Voss et al., 2011b), indicating the necessary role for hippocampus in these effects. The specific age-related deficits in the strategic enhancement of location recall and in revisitation therefore suggest that healthy aging could be accompanied by a reduction in hippocampal-prefrontal interactivity rather than an overall deficit in hippocampal function or in hippocampal interactivity with other brain regions. This novel hypothesis emphasizes the importance of hippocampal-prefrontal interactivity for effective learning (see also Chhatwal and Sperling, 2012; Sperling, 2007; Park and Reuter-Lorenz, 2009), and our paradigm for isolating strategies and their neural correlates could be useful in future experiments on neuropathological changes in healthy aging that specifically impair learning strategies as opposed to general memory function.

Finally, our approach for studying age-related changes in learning strategies and memory performance is consonant with a recent emphasis on the need to enrich translation of aging research from human to animal models (Alexander et al., 2012). In general, it is difficult to establish coherence between findings in humans and in animal models when tests involve words, verbal self-report, and/or semantic encoding strategies (as is the case in most previous studies on memory and in all previous studies on age-related changes in learning strategies). Our paradigm and results therefore make an important step towards development of across-species links in studies of aging and learning, given that similar exploration tasks can be implemented in rodents and primates (Metcalfe and Jacobs, 2010; Mata et al., 2013). Indeed, providing active control modulates representational qualities of hippocampal neurons in rodents relative to passive movement (Song et al., 2004), indicting that similar yoking procedures could be used in such translational studies of aging. By identifying specific age-related changes in learning strategies as well as the disruptions in neurological function that underlie them, it will be possible to enrich understanding of the challenges experienced during normal aging, whereas enhanced linkage to animal studies could ultimately provide neurobiologically motivated interventions.

## AUTHOR CONTRIBUTIONS

Both authors, Kelly L. Brandstatt and Joel L. Voss, performed substantial contributions to the conception of design of the work, acquisition, analysis, and interpretation of the data. Both authors drafted and revised the work critically for important intellectual content and have given final approval of the version to be published. Both authors are in agreement to be accountable for all aspects of the work in ensuring that questions related to the accuracy or integrity of any part of the work are appropriately investigated and resolved.

## Conflict of Interest Statement

Neither the authors nor Northwestern University received payment or services froma third party for any aspect of the submittedwork. There were no actual or potential conflicts of interest associated with this project. Appropriate approval and procedures were used concerning human subjects.
